# Cognitive functioning, sleep quality, and work performance in non-clinical burnout: The role of working memory

**DOI:** 10.1371/journal.pone.0231906

**Published:** 2020-04-23

**Authors:** Dela M. van Dijk, Willem van Rhenen, Jaap M. J. Murre, Esmée Verwijk

**Affiliations:** 1 Department of Psychology, University of Amsterdam, Amsterdam, The Netherlands; 2 Arbo Unie, Occupational Health and Safety Service, Utrecht, The Netherlands; 3 Department of Medical Psychology, Amsterdam University Medical Center, Amsterdam, The Netherlands; Imperial College London, UNITED KINGDOM

## Abstract

We investigated the relationship between cognitive functioning, work performance, and sleep in non-clinical burnout. In a working population, an online survey was conducted with additional online neuropsychological tests of varying complexity, measuring attention and different components of working memory, of which the coordinating subcomponent the ‘Central Executive’ is thought to be the most vulnerable to stress. Results indicate that non-clinical burnout is associated with more—though not severe—sleep problems, more depressive complaints, impaired work performance, and with both subjective and objective cognitive impairments. Compared with healthy respondents (*N* = 107), people with non-clinical burnout (*N* = 17) had a significantly poorer performance on the tests of the visuospatial sketchpad and the Central Executive of the working memory. Our study also indicates that more complex tests may be more sensitive in detecting cognitive dysfunction in non-clinical burnout. Furthermore, a relationship was found between dual-task performance and work performance. Regarding to sleep quality, in our sample of people with non-clinical burnout, there were no severe sleep problems. In the entire sample, however, insomnia was significantly related to subjective, but not objective, cognitive functioning, and also not to work performance.

## Introduction

‘*Mr. P. is a 54-year old consultant in a large international company. For almost a year he has had complaints of emotional and physical exhaustion, poor sleep, and other stress-related complaints. He also has difficulty concentrating, memorizing, and task-switching. He notices that his work performance is increasingly deteriorating. Finally, he reports sick and a burnout is diagnosed. Could this situation have been detected earlier? Would Mr. P.'s cognitive complaints already have been measurable in the non-clinical phase? How did his sleep complaints and cognitive problems influence his work performance?*’

### Burnout, prevalence, and definition

The prevalence of burnout is high. Since 1997, every year, a large-scale survey among Dutch employees is performed by the government (Nationale Enquête Arbeidsomstandigheden, NEA, by TNO & CBS). The prevalence of burnout symptoms in the Netherlands has increased dramatically: from 8% in 1997 to nearly 16% in 2017 [1]. In 2017, absenteeism due to stress, including burnout, cost Dutch employers an estimated 1.8 billion euro [1]. Comparison of burnout data between countries is difficult, since different diagnostic criteria and measuring instruments are used. Recently, however, an analysis of differences in burnout in European countries was conducted [2], based on data of the 6th European Working Conditions Survey (EWCS, 2015) among nearly 44,000 employers in 35 European countries. Prevalence of burnout was based on one of the items of the Maslach Burnout Inventory (MBI):*‘I feel exhausted at the end of a working day’*. When using the rather strict criterion of the maximum score (‘*always’* feeling exhausted) burnout percentages had a range from 4.3% (Finland) to 25% (Turkey), the Netherlands scoring in the low range–in contrast with the NEA questionnaire—with 6.4%. This lower percentage reflects a stricter criterion for inclusion.

Cognition has recently become central to the definition of burnout. Burnout is usually defined as a chronic strain response to (often work-related) stressors with three dimensions: emotional and physical exhaustion, depersonalization, and reduced personal/professional accomplishment [3]. A new definition of burnout has been proposed by Desart, Schaufeli & De Witte [4], based on interviews with general practitioners, occupational health officers, and psychologists. Besides exhaustion and depersonalization, two new core symptoms are included: Cognitive and emotional loss of control. These core symptoms, combined with secondary symptoms (depressive mood and stress symptoms), will ultimately lead to inefficacy. The addition of cognitive loss of control to the definition of burnout is understandable since many individuals with burnout report difficulties in their cognitive performance such as concentration and memory disturbances [5–8].

### Non-clinical burnout

In this study, we focus on impairments in cognition and sleep quality in the early phase of burnout and how this affects work performance. This early stage is usually called non-clinical burnout, as opposed to clinical burnout. Research has shown that the dimensions of burnout develop consecutively, at first with exhaustion and depersonalization. After some time, prolonged exhaustion may lead to problems with functioning [3, 7]. In non-clinical burnout, individuals have relatively mild burnout symptoms and are still working; it is imperative that they are detected in this phase, so that adequate preventive intervention can take place. After all, burnout may lead to health problems in the long-term, sometimes with long-lasting impairment of cognitive functions [9] or even irreversible cerebral damage [10].

### Cognitive functioning in burnout: The role of working memory

Though subjective cognitive dysfunction is often reported in burnout, remarkably few studies have examined the relationship between burnout and objective cognitive functioning, especially compared with the extensive research into burnout and, for instance, work performance. In 2014, Deligkaris, Panagopoulou, Montgomery, & Masoura reviewed the literature on cognitive performance during burnout in the period of 2005–2013 and were able to include only 15 studies [11]. They concluded that burnout patients tend to have impairments in the cognitive domains of attention, memory, and executive functioning. Later studies confirm this evidence for cognitive impairment in burnout [6, 11, 12]. When discussing the mechanism behind the association between burnout and cognitive functioning, they suggest that working memory could be the key component underlying the function of these three cognitive domains. Of the four components of the working memory [13, 14], the coordinating subcomponent the ‘Central Executive’ is thought to be the most vulnerable to stress compared with the three slave-systems that only store information (‘phonological loop’, ‘visuospatial sketchpad’ and ‘episodic buffer’) [11]. Summarizing, objective cognitive dysfunction in clinical burnout is well established by now, though many questions remain about the specific processes affected.

### Cognitive functioning in non-clinical burnout

The findings in behavioral studies of cognitive functioning in non-clinical burnout are less clear than in clinical burnout. Some studies find no cognitive impairment [15, 16] or even improved cognitive functioning [17]. Four studies, however, do find evidence for cognitive impairment in non-clinical burnout [18–21] with emerging evidence towards deficits in prefrontal cognitive functions, that is, executive functioning and working memory. Here, it must be kept in mind that executive functioning is also the most studied cognitive domain, making it more likely to be detected when dysfunctioning.

Results from recent physiological and brain-imaging studies into non-clinical burnout are in line with these findings of the involvement of prefrontal cognitive functions. EEG studies indicate impairment in executive functioning in non-clinical burnout (reduced amplitude of the P300), for example, in attention shifting [22, 23] and in response inhibition [24]. Clinically, people with such an impairment may be more easily distracted. A recent study suggests that reduction in brain-derived neurotrophic factor (BDNF) is associated with burnout as a result of inhibition of the HPA axis, which after years of chronic stress can lead to impaired neurogenesis and eventually neuron-atrophy [25]. In a large non-clinical burnout population, these reduced levels of BDNF were indeed found and appeared to have a mediating effect in the relationship between burnout and specifically prefrontal cognitive functioning (attention and immediate memory) [26].

Only one study on cognitive functioning in non-clinical burnout examined differences in task complexity in executive functioning tasks [18]. The authors found an association between high emotional exhaustion and reduced executive functioning but only in a condition that put high demands on executive functioning. This seems to indicate that tests of higher complexity are sensitive for detecting non-clinical burnout.

### Work performance

There is evidence that non-clinical burnout may impair work performance. A meta-analysis of 16 studies by Taris in 2006 showed negative correlations between exhaustion and objective measurements of work performance: in-role behavior, organizational citizenship behavior, and customer satisfaction [27].

Cognition may be impaired in non-clinical burnout and this likely contributes to impairments in work performance. Unfortunately, little research has been done on this topic. We were able to find only one study that investigated objective cognitive performance in relation to work performance [17] (in a population of working young adults). It concluded that examiner-rated lower occupational functioning was related to cognitive problems. More research has been done in cancer patients, albeit on subjective cognition, where studies have found that subjective cognitive problems exerts a negative influence on work performance [28, 29] and work productivity [30]. Based on these studies we expect that there may be negative effects of objective cognitive dysfunction in non-clinical burnout on work performance.

### Sleep

Besides cognitive dysfunction, also impaired sleep has been associated with burnout, both in clinical burnout [31, 32] and in non-clinical populations [33]. Grossi et al. conclude in their review that impaired sleep is one of the best predictors of burnout [34]. Impaired sleep is also associated with reduced work performance. Presenteeism (coming to work while sick, or suffering other conditions that prevent working productively) appears to be a significant problem in employees with insomnia [35] with an estimate of almost 8 days of lost work performance per year [36]. Though cognition and sleep have both been found to affect work performance, the mechanisms that govern these interactions are not clear at the moment.

Findings in studies on the association between impaired sleep and cognition appear inconclusive. There is ample support for a relationship between insomnia and subjective cognitive impairment in daytime functioning with, for instance, reduced work performance [37, 38]. Objective measurement of cognitive functioning in insomnia, however, gives conflicting results. A meta-analysis of these studies in 2012 with conflicting findings shows significant, though small-to-moderate, impairments in tasks assessing episodic memory, working memory, and problem solving [39]. More recent large studies, however, continue to have conflicting findings, showing evidence [40] and no evidence [41] of objective cognitive impairment in insomnia. The largest cross-sectional study thus far, involving 477,529 participants, even showed a surprising enhancement in objective cognitive functioning in patients suffering from insomnia [42]. This was also seen in the study of Altena et al.[43]. Some of the points of discussion regarding these conflicting findings concern the ecological validity of neuropsychological tests [39], the (low) complexity of the tests used [42], and personality factors in insomniacs (i.e. high achievers [43]).

### Summary

In summary, there seems to be an association between non-clinical burnout, work performance and sleep. It is not clear yet what is the role of cognition in this context.

**Fig 1:**
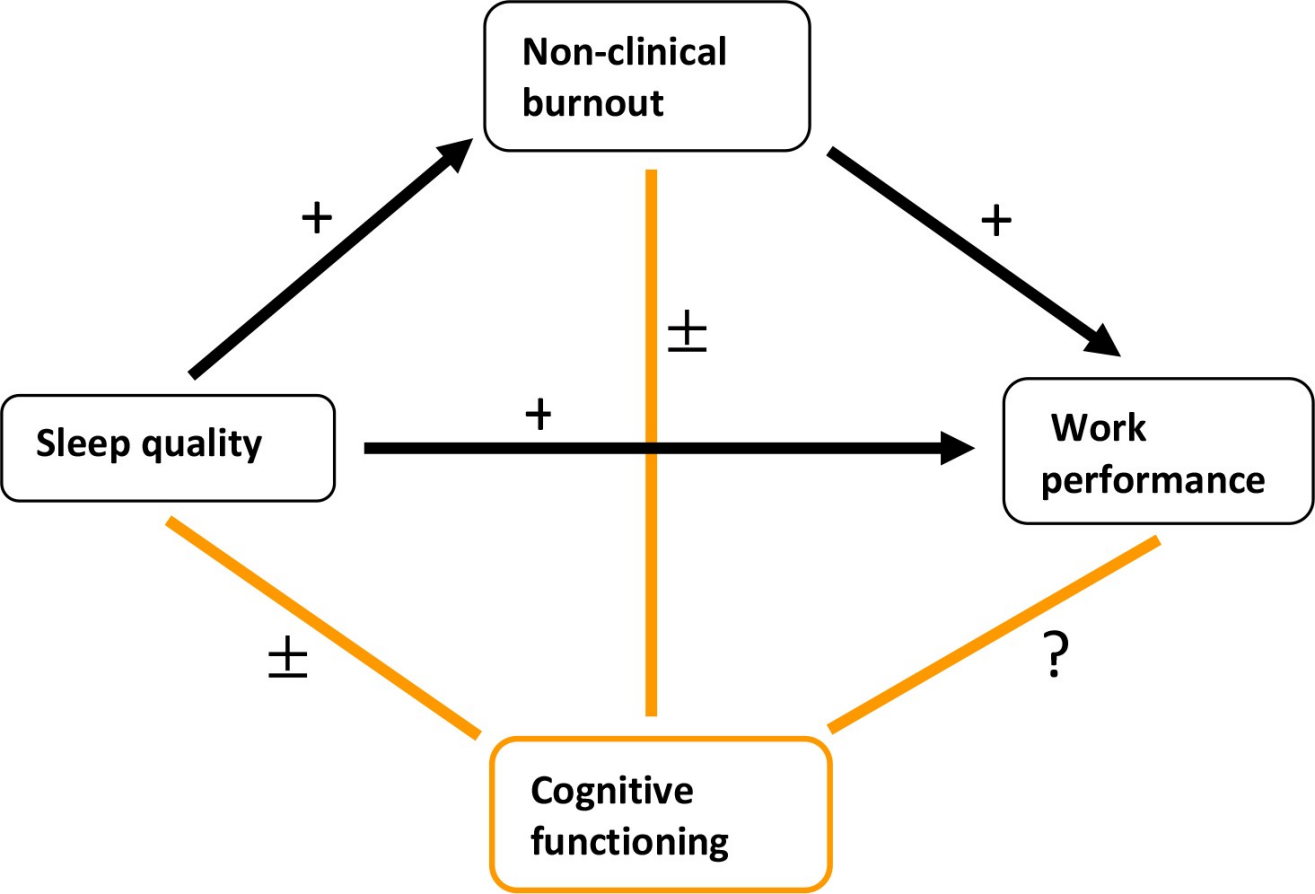
Diagram of the findings in the literature of non-clinical burnout, showing that much remains inconclusive or unknown regarding the relationship between cognitive functioning and non-clinical burnout, sleep quality and work performance. + = strong evidence, ± = inconclusive, ? = (almost) no studies.

Findings in the literature have strong evidence regarding the association between (see Fig 1):

1. Non-clinical burnout and reduced work performance
2. Impaired sleep and non-clinical burnout
3. Impaired sleep and reduced work performance
  Inconclusive or not yet investigated are the associations between:
4. Cognitive functioning and non-clinical burnout
5. Cognitive functioning in non-clinical burnout and work performance
6. Cognitive functioning and sleep quality

No study thus far seems to have investigated the interrelationship of all four of the above-mentioned variables: non-clinical burnout, cognitive functioning, work performance, and sleep quality. From a preventive point of view, it is worthwhile to investigate whether objective cognitive impairments contribute extra to early detection beyond predictive factors already known, such as sleep. Thinking back to our case study of Mr. P., we might wonder how his cognitive complaints could have been detected in the non-clinical phase. If successful, this may have paved the way for an effective intervention. To achieve this, two aspects are important: (1) early detection and (2) preventive intervention. (1) If (objective) cognitive functioning is impaired in non-clinical burnout, this may help in early detection of non-clinical burnout. (2) To clarify what the most effective preventive intervention should focus on, it is important to gain further insight into the interaction between cognition, sleep and work performance in non-clinical burn-out.

This study investigates whether cognitive functioning is impaired in non-clinical burnout and—if so—how this can be detected as early as possible and how this is related to job performance and sleep quality. We hypothesize that individuals with non-clinical burnout will indeed have lower subjective and objective cognitive functioning and that neuropsychological tests of higher complexity will be more suitable in detecting objective cognitive dysfunction. We also expect impaired cognitive functioning to be related to job performance and sleep quality.

## Materials and methods

### Ethics

The study protocol was approved by the Ethics Committee of the Department of Psychology of the University of Amsterdam.

### Participants

The participants in this study were employees of a Dutch business service-provider with 822 employees. The bulk of our sample were consultants, working with clients. Other positions included administrative support (like planning), a call-center, and staff positions. In most of these positions, employees have to switch much between different tasks (e.g., multiple consultations with clients or phone-conversations with customers).

Exclusion criteria were a) current sick leave, b) current or previous depression and burnout, c) medication or physical illness causing cognitive impairment, such as benzodiazepines or thyroid disease, d) alcohol or substance abuse, and e) visual impairment.

### Procedure

All employees were invited by email to participate in this study, which included a link to the website where the questionnaires (self-reports) and neuropsychological tests were programmed (https://scripting.neurotask.com/, [44]). A reminder email was sent twice with a two-week interval. Before starting, the participants received information about the procedure and confidentiality of the research and they stated their Informed Consent. Participation was voluntary. Participants received no reward.

Subjects were tested online and the tests concluded with an automatized feedback with advice to consult a doctor when respondents scored outside cut-off points for clinical disorders (see below). The researchers did not have access to this feedback.

### Questionnaires

First, general questions were asked about demographic variables (age, gender, and education), use of medication, alcohol, or drugs, and current or previous illness that could influence cognition.

#### Burnout

The Burnout Assessment Tool (BAT) [45] was used to distinguish between people with non-clinical burnout and healthy people. This self-report questionnaire has been developed recently, based on the new definition of burnout and an analysis of the various existing burnout questionnaires. It is currently being validated in different countries. It consists of 34 items asking employees how often they experience certain problems or complaints. The items are distributed over six scales: Exhaustion (8 items, i.e. ‘At work, I feel mentally exhausted’), Mental Distance (5 items, i.e. ‘I feel indifferent about my job’), Emotional Impairment (5 items, i.e. ‘At work, I feel unable to control my emotions’), Cognitive Impairment (5 items, i.e. ‘At work, I have trouble staying focused’), Psychological Complaints (6 items, i.e. ‘I tend to worry’), and Psychosomatic Complaints (5 items, i.e. ‘I suffer from palpitations or chest pain’). Response options range from 1 = ‘never’ to 5 = ‘always’. The BAT has good internal consistency (.87 - .97 for the different scales) and factor validity [45]. After consulting with the founder of the questionnaire, the cut-off point for non-clinical burnout was set on the 75^th^ percentile of a representative sample of 1500 Dutch employees, which corresponded to a score of 83 [46]. As the cut-off point for clinical burnout, the average score of a Flemish patient group was used, which corresponded to a score of 111. Above this cut-off point for clinical burnout, respondents in our study received advice to consult a doctor.

#### Depression

The depression scale of the Four-Dimensional Symptom Questionnaire (4-DSQ) [47] was used to investigate possible depressive symptoms. The 4-DSQ is a self-report questionnaire that has been developed in primary care to distinguish non-specific general distress from depression, anxiety, and somatisation. It has proven to be a reliable and valid instrument in primary care [48] but also in a working population [49]. The depression scale consists of 6 items asking people how often a certain symptom has been experienced (i.e. ‘do you feel that life is not worth living’). The reference period is ‘the past week’. The response categories are ‘no’, ‘sometimes’, ‘regularly’, ‘often’, and ‘very often or constantly’. The responses are scored as 0 for ‘no’, 1 for ‘sometimes’, and 2 for the other response categories. The item scores are summed to the scale score. A score of 0–2 is considered as ‘probably no depressive disorder’, a score of 3–5 as ‘possible depressive disorder’ (re-evaluation is advised after a few weeks), and 6–12 as ‘relatively high risk of a depressive disorder’. Respondents in our study who scored in the highest range received advice to consult a doctor and were excluded.

#### Subjective cognitive functioning

The Cognitive Symptom Checklist-Work Dutch Version (CSC-W DV) is the Dutch translation of the CSC-W21 [50], a brief self-measure of cognitive symptoms in the context of work, developed in a group of breast cancer survivors. The original, unabridged, questionnaire CSC is a generic self-report questionnaire. Although the CSC-W21 (and CSC-W DV) has not been used in populations other than cancer patients, we think the questionnaire can be used in the general working population as well, since there are no questions that are specific to the situation of cancer patients. The CSC-W DV has good validity and high reliability (Cronbach’s *α* = 0,93–0,95) without floor or ceiling effects [28]. It contains 19 items asking employees about problems they might experience in their work situation. The items are distributed over two scales: working memory (8 items, for example ‘I have difficulty remembering my train of thought as I am speaking’) and executive functioning (11 items, for example ‘I have difficulty understanding how a task fits into a plan or system’). Response options range on a five-point scale from 0 = ‘none’ of the time to 4 = ‘all the time’. Total scores are calculated by summing all scores, divide them by the number of items, and then multiplying them by 25. Total scores range from 0 to 100, with higher scores indicating more work-specific cognitive symptoms. CSC-W scores appear to be related with work productivity [30] and work functioning [28].

#### Work performance

The Individual Work Performance Questionnaire (IWPQ) is an 18–item self-report questionnaire with questions concerning employee’s behavior at work in the past three months [51]. Items are distributed over 3 scales, representing the underlying three-dimensional conceptual framework: Task Performance (5 items, i.e., ‘My planning was optimal’), Contextual Performance (8 items, i.e., ‘I took on extra responsibilities’) and Counterproductive Work Behavior (5 items, i.e., ‘I made problems greater than they were at work’). Each item is rated on a 5-point scale ranging from 1 = ‘seldom’ to 5 = ‘always’. The questionnaire has good psychometric characteristics with, for instance, a good reliability (Cronbach’s *α* ranging from 0.78–0.85 for the different scales) [51].

#### Sleep quality

The Dutch translation of the Insomnia Severity Index (ISI) [52] measures sleep quality. This brief self-reported instrument is a widely used instrument of adequate reliability (Cronbach’s α = 0.78; average item-correlations = 0.54). It contains 7 items assessing the severity of initial, middle, and late insomnia; sleep satisfaction; interference of insomnia with daytime functioning; whether sleep problems are noticed by others; and distress about sleep difficulties. The items are scored on a 5-point scale (0–4) with a maximum total score of 28. A score of 0–7 is considered as ‘no insomnia’, 8–14 as ‘sub-threshold insomnia’, 15–21 as ‘moderate insomnia’, and 22–28 as ‘severe insomnia’. When scoring within the highest range, respondents in our study received advice to consult a doctor.

### Neuropsychological tests

Tests of increasing complexity were used to assess attention and working memory, including the Central Executive component of working memory. To our knowledge, this has not been done before in published studies. With tasks of higher complexity, we mean tasks that are cognitively more demanding. We consider a single attention task to be of relatively low complexity. Dual-tasks are generally seen as cognitively more challenging.

Findings in the literature suggest deficits in prefrontal cognitive functions in non-clinical burnout, in particular in executive control and working memory. Deligkaris et al. [11] suggest that the Central Executive is most vulnerable to stress. It is, therefore, worthwhile to explore the functioning of the Central Executive. A common way to measure this is by dual-task performance [53], where typically two of the slave systems of the working memory, phonological loop and the visuospatial sketchpad, are strained simultaneously [54]. The *n*-back task is also is also thought to strongly involve the Central Executive (in particular the updating component) [55].

The tests are summarized in Table 1 and described in more detail below. Respondents were instructed not to perform the tests on a tablet or mobile phone, as this might give different test results, compared with performance on the PC. Each test was preceded by an instruction and a practice trial with automatically generated feedback when making a mistake. For instance, the following feedback could be given: *‘Make sure that you click on the numbers in the same order as shown above’*.

**Table 1:** Neuropsychological tests.

↑ *higher complexity*			Dual Task *n*-back 1 and 2 (updating)
		Digit Span Sequencing (phonological loop) Corsi Block (visuospatial sketchpad)	
	Digit Span Forward		
	**Attention**	**Slave Systems Working Memory**	**Central Executive Working Memory**

### Attention

We used the Digit Span Forward as a test of attention. as suggested by Lezak, Howieson, and Loring [56].

**Fig 2:**
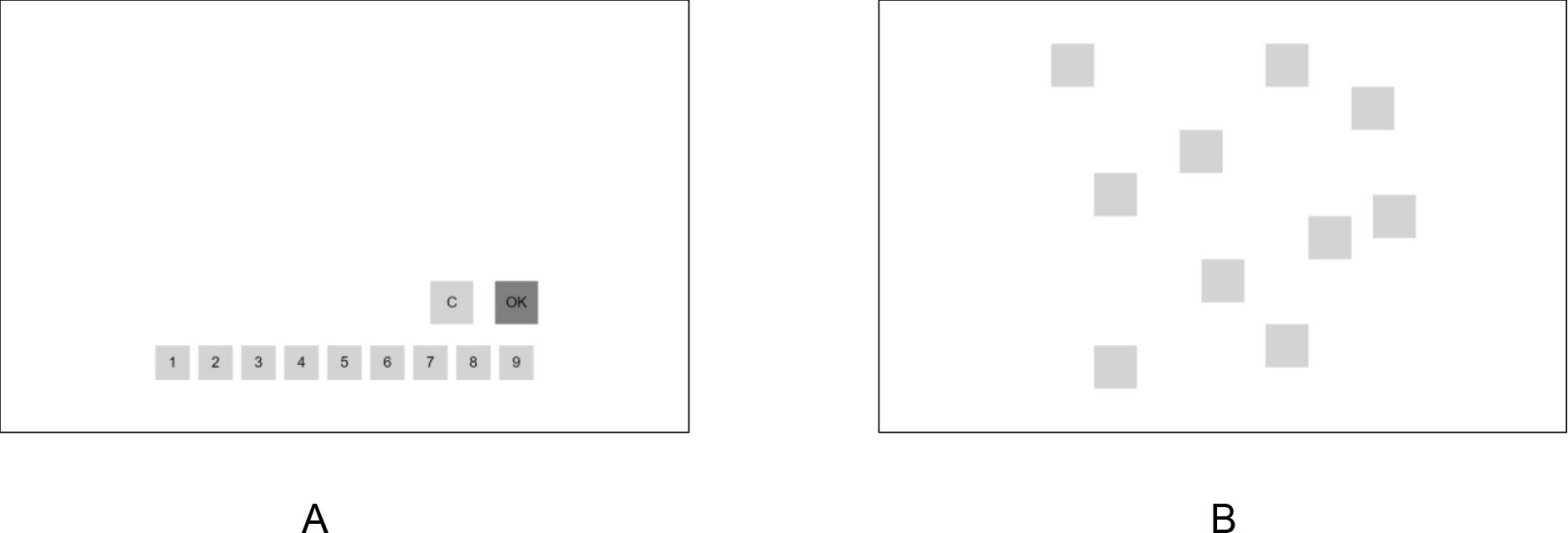
Examples of two online tests. (A) Response page of the Digit Span Tests. (B) Layout of the blocks in the Corsi Blocks test.

For decades, digit span tests have been part of the Wechsler Adult Intelligence Scale (WAIS). For our study, we used the WAIS-IV-NL [57]. The Digit Span Forward test contained a maximum of 16 trials, each consisting of a sequence of digits, which were displayed in a quasi-random order. All respondents received the same trials. Each digit was presented for 1000 ms with an interval of 500 ms. A sequence of digits could consist of the numbers 1 to 9, in which a particular digit could be presented more than once. The number of digits in a sequence increased after every two trials, beginning with a sequence of two digits. Participants were instructed to recall the digits in the order in which they had been presented. They had to click on the corresponding digits that were displayed in boxes at the bottom of the screen (see Fig 2A). Participants could correct an error and while completing a sequence. They clicked the ‘OK’ button to signal completion of the response, which also started the next trial. After clicking the ‘OK’ button, it took 1000 ms before the next sequence of digits was presented. The test was aborted when two trials of a particular length were recalled wrongly (each containing at least one mistake). The length of the last sequence that was recalled correctly was taken as the span. The outcome of the test was measured with the product score, being the number of correct recalled sequences times the span.

#### Slave systems of the working memory

The Digit Span Sequencing was used for testing the phonological loop of the working memory.

The test was carried out in the same way as the Digit Span Forward, as described above, but respondents now had to recall the digits in ascending order, including repetition of any digits that were included twice.

The Corsi Block test was used for testing the visuospatial sketchpad. It was an adaptation of the widely used Corsi Block Tapping test [58–61] and also contained a maximum of 16 trials. At the start of the test, nine grey colored spatially separated blocks were shown on the screen on a white background. The size of the blocks was 10 by 10 percent windows height (see Fig 2B). On each trial, every 1000 ms, a sequence of squares briefly changed color to black (‘blink’) during 500 ms. At the end of a sequence, the white background would briefly change color to light yellow, indicating that the sequence was finished. The respondents then had to click on the blocks in the recalled spatial locations and in the same order as was shown to them. After finishing doing so, the next trial would automatically start. The other details of the procedure and outcome measure were the same as for the digit span tests.

#### Central executive of the working memory

For testing the Central Executive, we combined the Digit Span Sequencing and the Corsi Block Test, with digits appearing in the blocks. Respondents had to click on the blocks in which they recalled seeing the digits, again in ascending order. That is, they had to ignore the order in which they had appeared in the blocks. We called this test the Dual Task.

Combining the Digit Span Sequencing and the Corsi Block Test has been described before by Towse and Houston-Price [62]. Their implementation differs from ours in three ways: (1) they used the Digit Span Forward, (2) our respondents made the tests alone in an unmonitored setting, and (3) their respondents had to point at the blocks, while verbally reporting the digit they had seen in them. For reasons mentioned above, we chose the Digit Span Sequencing instead of the Digit Span Forward as a test for the phonological loop and, therefore, we consider our version of this dual task test a more accurate way of straining the Central Executive. Although this test as a whole has not been validated, both underlying tests are well validated. Therefore, there seemed to be no need for an extensive validation. Before starting the study, three volunteers piloted the tests to verify whether the instructions were sufficiently clear. No floor or ceiling effects were found for the Dual Task.

The *n*-back test, first described by Kirchner [63], is widely used in studies on working memory. Although not suitable for measuring individual differences, due to its low reliability, the *n*-back test has proven to be useful for experimental research [55]. The test consisted of 40 letters, which were presented one by one in the center of the screen during 500 ms with a fixed inter-stimulus interval of 2500 ms. Stimuli consisted of the letters C, F, B, Q, S, W, J, D, M, V, and F, which were displayed in a quasi-random order in capital letters. Respondents were instructed to press either the spacebar or instead click with the mouse on a ‘YES’ button when the displayed letter was identical to the letter, or do nothing otherwise. Identical letters were displayed one stimulus earlier (e.g. W-W-V, *n*-back 1) or two stimuli earlier (e.g. F-C-F, *n*-back 2) quasi-randomly with a target rate of 33%. The maximum response time was fixed at 1200 ms. The number of correct scores minus the false alarms was used as an outcome measure.

### Statistical analyses

To test for differences in means of all variables (results on all questionnaires and test scores) between the non-clinical and healthy group we used t-tests or Mann-Whitney U tests in case of non-normality. A Benjamini and Hochberg correction was performed for multiple testing [64].

To examine if individuals with non-clinical burnout have a relatively poorer performance on tests with higher complexity compared with healthy individuals, a Mixed ANCOVA was performed with age and education as covariates. The within-subject variable was ‘Working Memory tests’ with two levels: ‘Slave systems’ (Level 1, lower complexity) and ‘Central Executive’ (Level 2, higher complexity). The level ‘Slave systems’ consisted of the sum score of the standardized scores on the Corsi Block Test and the Digit Span Sequencing. The level ‘Central Executive’ consisted of the sum score of the standardized scores on the Dual Task and the two *n*-back tasks. The between-subject variable was the score on the burnout questionnaire, the ‘BAT score’, with two conditions, the healthy group (condition 0) and the non-clinical burnout group (condition 1).

To further elucidate the relations between non-clinical burnout, cognitive dysfunction, perceived sleep quality and work performance, a correlation analysis was done for the non-clinical burnout group in which all the above-mentioned variables were included. If relevant, a subsequent regression analysis was performed.

All statistical results were based on an alpha level of 0.05. The data analysis was performed using IBM SPSS Statistics 25.

## Results

There was a response of 17%, 141 respondents out of 822, of whom 17 had to be excluded. 15 participants met 1 or more of the exclusion criteria. 1 respondent was excluded because of failing to register age, 1 because of an obviously wrongly registered age (18 years with a college degree), leaving a total of 124 respondents. Extreme scores found through visual inspection of the test score distributions were investigated in more detail. This led to the removal of 8 test scores, usually, when it was clear that a respondent had not understood the instruction (e.g. when also the example was done wrong). Demographic characteristics are listed in Table 2. The data are accessible online at https://osf.io/vg4c8/.

**Table 2:** Demographic characteristics of the respondents (N = 124) and the organization (N = 822).

		Respondents	Organization
Gender (%)	Female	62.9	61.4
	Male	37.1	38.6
Age (years)	Mean	51.3	51
Education (%)	Verhage 5	13.7	
	Verhage 6	34.7	*Unknown*
	Verhage 7	51.6	

Verhage educational category: 5 = finished average-level secondary education, 6 = finished high-level secondary education, and 7 = university degree [65].

**Fig 3:**
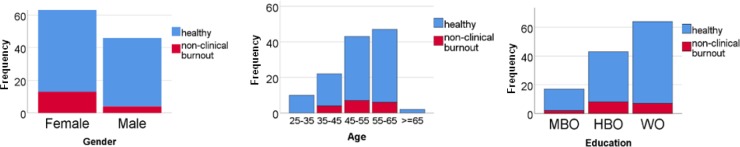
Demographic characteristics of the respondents (*N* = 124) with a comparison between the healthy (*N* = 107) and the non-clinical burnout (*N* = 17) group.

14% of the respondents met the criteria for non-clinical burnout (17 respondents, 76.5% female, 23.5% male, mean age 50.9 years). Comparison of the non-clinical burnout and the healthy group (107 respondents, 60.7% female, 39.3% male, mean age 51 years) (see Fig 3) revealed no significant difference in age, *U* = 808, *z* = 0.74, *p* = .46, or education, *U* = 824.5, *z* = 0.68, *p* = .50. A Chi-Square test with Yates’ correction on the 2x2 contingency table showed no significant difference in gender, χ^2^(1) = .95, *p* = .33.

A Mann-Whitney U test showed a significantly poorer performance in the non-clinical burnout group compared with the healthy group in subjective cognitive functioning and on two of the six objective tests, namely the Corsi Block Test and the Dual Task (see Table 3). The results of these two tests remained significant after correction for multiple testing, yielding *p* = .045 for both tests. Compared with healthy individuals, respondents with non-clinical burnout also reported significantly more sleep problems though not severe (‘sub-threshold insomnia’ versus ‘no insomnia’), depressive complaints (but still below the cut-off for depressive disorder), and impairments in their work performance.

**Table 3:** Comparison of cognitive performance between the healthy (N = 107) and the non-clinical burnout (N = 17) group.

Variable	*Mdn (SD)* non-clinical burnout group	Range	*Mdn (SD)* healthy group	Range	*U*	*z*	*p (one-tailed)*
Questionnaires							
BAT	92.0 (8.88)	84–117	62.0 (12.6)	34–82			
CSC-W DV	43.0 (7.48)	32–59	37.0 (7.18)	19–51	399	3.71	< .001
ISI	13.0 (1.62)	0–21	5.00 (3.94)	0–15	224.5	4.99	< .001
4 DSQ depression	2.00 (6.73)	0–5	0.00 (0.75)	0–3		4.16	< .001
IWPQ	30.0 (6.73)	14–39	33.0 (7.40)	19–56	478.5	1.82	.034
Tests					659.5		
Digit Span Forward	48.0 (18.4)	24–88	54.0 (28.1)	12–144	799	0.81	.21
Digit Span Sequencing	70.0 (24.2)	40–126	73.5 (27.6)	12–135	892.5	0.063	.47
Corsi Block Test	37.5 (12.8)	20–77	40.0 (19.8)	9–104	556.5	2.11	.017
Dual Task	30.0 (19.7)	6–77	48.0 (21.5)	9–112	577.5	2.42	.007
*n-*back 1	12.0 (1.54)	6–12	12.0 (1.52)	5–12	751	0.76	.22
*n-*back 2	9.00 (2.65)	2–12	9.00 (2.30)	3–12	841.5	0.44	.33

BAT = Burnout Assessment Tool, CSC-W-DV = Cognitive Symptom Checklist-Work-Dutch Version, ISI = Insomnia Severity Index, 4-DSQ = Four Dimension Symptom Questionnaire, IWPQ = Individual Work Performance Questionnaire.

The Mixed ANCOVA revealed a main effect of the Working Memory tests (within subjects effect), *F*(1,114) = 8.82, *p* = .004, $\eta_{p}^{2}$ = .072. There was no main effect of the BAT score (between subjects effect), *F*(1,114) = 2.76, *p* = .1, $\eta_{p}^{2}$ = .024. The predicted interaction effect between Working Memory tests and BAT score was significant, *F*(1,114) = 6.91, *p* = .01, $\eta_{p}^{2}$ = .057. These analyses included the effects of the covariates education and age. Of these covariates, only education significantly predicted test score, *F*(1,114) = 8.49, *p* = .004, $\eta_{p}^{2}$ = .069, and had a significant interaction effect with Working Memory tests, *F*(1,114) = 8.36, *p* = .005, $\eta_{p}^{2}$ = .068.

A correlation analysis of the non-clinical burnout group (see Table 4) showed that work performance was significantly correlated with subjective cognitive functioning and the Dual Task. Sleep quality was not related with any of the variables. Subsequent linear regression analyses confirmed this. The relation with work performance was significant for subjective cognitive functioning, *β* = -0.48, *t*(1,15) = 2.11, *p* = .031, and for the Dual Task, *β* = 0.51, *t*(1,15) = 2.29, *p* = .02.

**Table 4:** Correlations in the non-clinical burnout group (Spearman’s rho for Corsi Block Test and n-back1, Pearson’s r for the other variables).

Variable	CSC	ISI	4 DSQ	IWPQ	DSF	DSS	CBT	Dual Task	*n-*back1	*n-*back2
Questionnaires										
CSC-W DV										
ISI	.21									
4 DSQ depression	.20	.14								
IWPQ	-.48*	-.20	-.41							
Tests										
Digit Span Forward	.003	-.21	.40	-.33						
Digit Span Sequencing	-.25	-.19	-.24	-.26	.35					
Corsi Block Test	.58**	.095	-.050	-.42	.081	.26				
Dual Task	-.28	.17	-.25	.51*	-.45*	-.041	-.079			
*n-*back1	.065	.30	-.094	.063	-.55*	-.32	.16	.28		
*n-*back2	.011	-.073	-.048	.39	.068	-.099	-.22	.42*	-.20	

*N* = 17

* *p* < .05

** *p* < .01 (one-tailed).

BAT = Burnout Assessment Tool, CSC-W-DV = Cognitive Symptom Checklist-Work-Dutch Version, ISI = Insomnia Severity Index, 4-DSQ = Four Dimension Symptom Questionnaire, IWPQ = Individual Work Performance Questionnaire.

## Discussion

This study investigated the association between cognitive functioning, sleep quality, and work performance in non-clinical burnout. Compared with healthy individuals, respondents with non-clinical burnout reported significantly more sleep problems though not so severe that they would be qualified as insomnia. They also had more depressive complaints, but these remained well below the cut-off for depressive disorder. Furthermore, the non-clinical burnout group had more impairments in their work performance. Also, their cognition was affected.

People with non-clinical burnout, compared with the healthy group, had a significantly poorer performance in subjective cognitive functioning and scored significantly lower on two of the six objective tests, namely the tests of the visuospatial sketchpad (Corsi Block Test) and the Central Executive (Dual task performance) of the working memory. As expected, both groups performed lower on the more complex working memory tests measuring the Central Executive component compared with the less complex tests measuring the Slave Systems. However, the difference in performance on the more versus less complex tests was significantly larger in the non-clinical burnout group, compared with the healthy group. In the non-clinical burnout group, work performance was significantly correlated with subjective cognitive functioning and the Dual Task. Perceived sleep quality in non-clinical burnout was not significantly correlated with either cognitive functioning or work performance.

In our sample, only 14% of the respondents scored above the cut-off point for non-clinical burnout. This is lower than the expected 25%, since the cut-off point was based on the 75^th^ percentile of a representative sample of 1500 Dutch workers. Our sample, therefore, seems ‘healthier’ than expected. Two explanations for this difference can be given. First, since psychosocial work factors—such as workload, autonomy, and social support—are important in association with burnout [66], our findings might suggest a sample bias, where the working climate in the organization we invested is favorable regarding these work factors. Second, a less positive explanation could be that there was a selection bias, in which people with more burnout complaints did not participate, for instance because they did not have enough energy or motivation to make time for it.

### Cognitive functioning in non-clinical burnout

Our findings of impaired subjective cognitive functioning are in line with the results of all three studies we found on subjective cognitive functioning in non-clinical burnout [16, 67, 68]. This is, therefore, an extra justification for including impaired cognitive control in the new definition of burnout with the attendant development of the new Burnout Assessment Tool [4].

The poorer dual-task performance in non-clinical burnout—compared with healthy respondents—suggests the involvement of the working memory component Central Executive in non-clinical burnout, as we hypothesized, based on earlier suggestions in the literature [11]. The other tests for the Central Executive, the *n-*back tests (typically associated with the updating component of executive functioning [69]), however, did not reach statistical significance. This might indicate that dual-task performance is more vulnerable to chronic stress than the process of updating. We might explain this by differences in the underlying mechanism of the two tasks. The *n-*back test is based on a recognition-based process, whereas dual-task performance relies on active recall, the latter probably being more effortful. No firm underpinning could be found for this in the literature, but it is worthwhile to investigate this in further research.

The difference between the non-clinical burnout group and the healthy group in performance on the Corsi Block Test but not on the Digit Span Sequencing as tests for the Slave Systems of the working memory is noteworthy. A further explorative analysis for the entire sample gave the same result with overall significant poorer performance on the Corsi Block Test compared with the Digit Span Sequencing (*p* < .001). There are different possible explanations for this. First, one could conclude that verbal (working) memory tasks are easier than visuospatial tasks. This is, however, not in line with findings in the literature. On the contrary, verbal memory tasks are sometimes regarded as being more difficult than visual memory tasks [70]. This finding, however, is negated by a large online study in which more than 100.000 verbal and visuospatial memory single task sessions were performed, which revealed large individual differences in verbal versus visuospatial memory performance [71]. A second explanation could be found in the study of Murre et al. (2013), namely that after age 25, visuospatial memory decreases about twice as fast as verbal memory. Since the average age in their large sample was 37.4 compared with 51.6 in our sample, one could hypothesize that a correction for age in our study would give similar results. To test this hypothesis, we did a further analysis of our data by comparing two age groups, one group with an average age of 37.5, as in Murre’s study (all respondents were under age 45, *N* = 29) and an older group with an average age of 55.5 (all respondents 45 years and older, *N* = 95). The results were not in line with our expectations. Both age groups performed significantly poorer on the Corsi Block Test compared with the Digit Span Sequencing (*p* < .001 in both groups). When compared with the older group, the younger group performed significantly better on the Digit Span Sequencing (*p* = .05) with no difference in performance on the Corsi Block Test. For a third explanation, one could think of a response modality effect, since our stimuli were offered visually rather than verbally. Findings on response modality effects in serial recall are mixed. In general, an advantage for serial recall with verbal presentation is reported [72, 73]. Others, however, question this finding [74] and the study of Klinger, Tversky, and Hanrahan suggests a lower cognitive load for visual compared with verbal presentation [75]. Finally, one might question whether the Digit Span Sequencing is the best way of testing verbal working memory. According to Bouma, Mulder, Lindeboom, and Schmand, the Letter-Number Sequencing of the WAIS-IV is a more sensitive test for testing working memory, although others question this [66, 76].

Another remarkable result of our study was the fact that both the healthy and the non-clinical burnout group performed better on the Digit Span Sequencing than on the Digit Span Forward since we had the idea that the latter was less complex. Based on the feedback from the volunteers who piloted the experiment an explanation for this may be that the Digit Span Sequencing enables using memory strategies more than the Digit Span Forward. Oberauer, Lane, and Engle (2004) suggest that dual-task versions of serial order memory tasks are more difficult and a purer estimate of working memory since they prevent the use of strategies [77].

Our findings indicate that people with non-clinical burnout score relatively poorer on the more complex tests of the Central Executive, compared with the simpler tests of the Slave Systems, indicating that the more complex tests are more sensitive in detecting non-clinical burnout (hypothesis 2). This is in line with the findings of an earlier study that found poorer performance on tasks that put higher demands on executive control (i.e. more complex) compared with tasks that put lower demands on executive control [18]. It should be pointed out, however, that to compare the Slave Systems with the Central Executive, we combined the tests in a compound score in order to be able to compare two levels of complexity. Further explorative analyses did not reveal any difference when separating the tests. Therefore, no firm conclusions can be drawn, regarding the sensitivity of the individual tests.

### Work performance

The significant positive association found between work performance and subjective cognitive functioning in non-clinical burnout is not very surprising since we used the Cognitive Symptom Checklist–Work (Dutch version), which focuses on work-related cognitive complaints.

The positive relationship between work performance and objective cognitive performance on the Dual Task seems to be a robust finding, since we were already able to detect it in a relatively small non-clinical burnout group (*N* = 17). Remarkably, this relationship was not found in the much larger healthy group. The obvious assumption that the severity of burnout complaints underlies this correlation was positively confirmed in a further analysis of the entire group, which showed that the score on the burnout questionnaire (BAT) was significantly correlated with work performance and with the Dual Task (and none of the other tests). Based on this result, combined with the earlier suggestion by Deligkaris et al. [11] that impairments in working memory could be a mediating factor for the link between burnout and reduced work performance, we decided to run an additional mediation-analysis and moderation analysis for the entire group with work performance (IWPQ) as dependent variable and BAT and Dual Task scores as independent variables. No mediation effect or moderation effect, however, was found. The finding that the Dual Task was the only test that was significantly related to work performance, could perhaps be explained by the fact that dual-task performance is associated with task-switching [78, 79], an activity that people often engage in while working.

**Fig 4:**
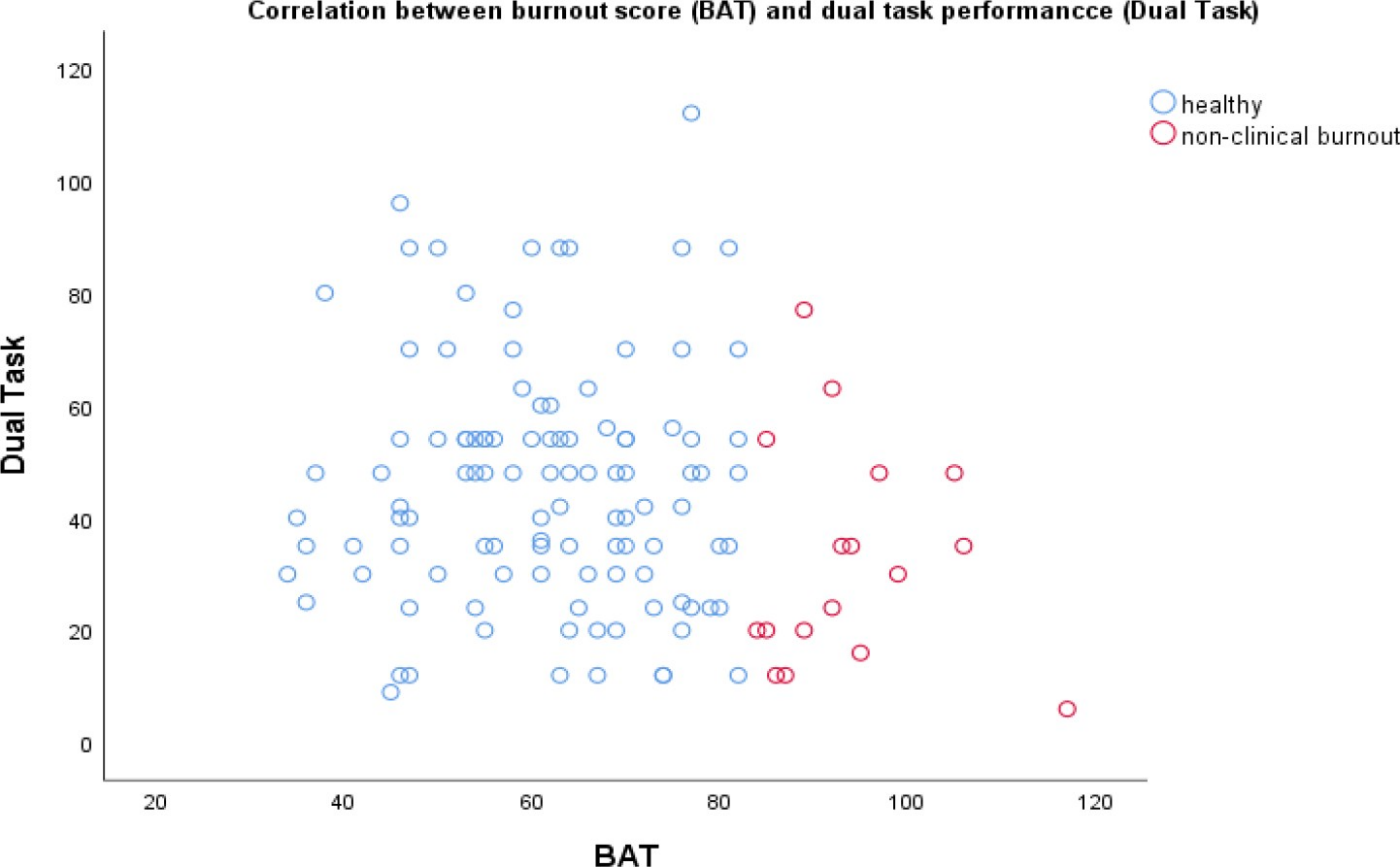
The relationship between dual-task performance (Dual Task) and burnout score (BAT) in the entire sample (*N* = 124).

The above-mentioned significant relationship between dual-task performance and work performance must be viewed with some nuance. Since we assumed a non-linear relation between severity of burnout and dual-task performance we made a scatter plot (see Fig 4). This showed that one very high burnout score (above the clinical cut-off point) with a very low test score appeared to be accountable for this relationship. After removing this data-point the relationship between BAT score and dual-task performance did not reach significance anymore. The Mann Whitney test between the non-clinical burnout and the healthy group was still significant for both the Corsi Block Test and the Dual Task test, but these results did not remain significant after correction for multiple testing. Although this data-point is a valid one, our findings are not as robust as they seem to be. Further research with larger samples will provide more reliable insight into this matter.

Surprisingly, people with non-clinical burnout with lower work performance had a higher score on the (three) tests with the lowest complexity compared with the healthy individuals (see Table 4) although this difference was not statistically significant. An explanation for this finding could be that people with non-clinical burnout who experience poorer performance in their work try harder to put a good performance on the tests. Certain personality traits, such as perfectionism, are risk factors for burnout [80] and could be underlying to this finding. When the tests become more difficult (the tests of the Central Executive), however, they can no longer compensate. Similar suggestions concerning personality traits are given in the study of Castaneda et al., who found enhanced cognitive functioning to be associated with higher burnout scores [17]. Similar findings regarding enhanced cognitive performance in ‘high achievers’—although with insomnia instead of burnout complaints—were described in the study of Altena et al. [43]

### Sleep

In our study, perceived sleep quality in non-clinical burnout was not correlated with either cognitive functioning or work performance. For the most part, this is not in line with findings in the literature. There is ample support for a relationship between impaired sleep and subjective cognitive performance [37, 38] and between sleep quality and work performance [35, 36]. An important explanation for this can be found in the fact that the mean score of the non-clinical burnout group was still in the range of sub-threshold insomnia. Indeed, further analysis of the entire sample revealed a significant difference in subjective cognitive functioning between individuals with insomnia (ISI score > 14, *N* = 9) and those without insomnia (ISI score < 8, *N* = 82). No difference in the entire sample was found regarding work performance. A significant difference in work performance was only found when comparing individuals with insomnia with the 25% best sleepers. In both these two further analyses, there was no significant difference in performance on any of the neuropsychological tests. Findings in the literature regarding impaired sleep and objective cognitive functioning are inconclusive as described earlier. The results of this study support those studies that also do not find a relation between impaired sleep and objective cognitive functioning (i.e.,[41]).

### Practical implications

The findings of this study are of practical relevance. After all, early detection of possible cognitive dysfunction in non-clinical burnout is important, not only from a health preventive view, as explained earlier, but also from a business-economic point of view. The Dual-Task used in our study could serve as the starting point to develop a more sensitive diagnostic instrument that can detect those cognitive impairments relevant for the early detection of non-clinical (and probably also clinical) burnout. Interventions in the field of temporary adjustments in work for people with non-clinical burnout could be used to prevent future absenteeism. This should include, in particular, reducing task-switching by offering more simple and well-defined tasks.

Regarding potential treatment of cognitive deficits, findings are inconclusive. Several studies have focused on the training of working memory and executive functions and many found promising results. In their meta-analysis, however, Melby- Lervåg and Hulme, conclude that these training programs produce only short-term, training-specific, effects that do not generalize [79]. These studies did not focus on (non-clinical) burnout, however. The first study to investigate a cognitive training intervention in stress-related exhaustion did find evidence of generalization effects of the trained task with considerable effect sizes (Cohen’s *d* between 0.40 and almost 1.0) [81]. This only applied to untrained tasks that targeted the same abilities (near transfer), however, and not to tasks measuring other, also untrained, abilities (far transfer). Interestingly, they found a significantly larger reduction in subjective cognitive complaints with a medium effect size (Cohen’s *d* = 0.70) and levels of burnout in the training group compared with the treatment as usual group. The latter findings suggest that cognitive training in burnout might be a valuable intervention.

### Limitations

As a first limitation, in order to keep the test session brief and thus guarantee enough participants, we decided not to include shifting tasks and inhibition tasks in the study. In our original design, we intended to include a version of the Dual Task with a shifting condition, an inhibition condition, and a combined shifting and inhibition condition. This would have enhanced the complexity of the tests and completed the test battery with all of the three basic executive functions—updating, shifting and inhibition—in line with the model by Miyake et al. [69] and might have given us a deeper insight into which complex tests are most sensitive in detecting non-clinical burnout.

Second, as already discussed earlier, the way we measured verbal working memory might be improved with a more sensitive test. In retrospect, it might have been better to have used the Letter-Number Sequencing test instead of the Digit Span tests.

Third, where cognitive functioning was determined both subjectively and objectively, this was not done for sleep. This was not feasible logistically, but could have provided further insight into the role of sleep.

Fourth, since this study has a cross-sectional design, no firm conclusions can be drawn on the cause-effect relationship between the associated variables.

Fifth, the sample of people with non-clinical burnout was small. On the one hand, this makes generalization to the population less reliable. On the other hand, given that the statistical significance was already found in such a small sample, this suggests that more sensitive tests may be developed that can be used with individual subjects rather than be applied only at the group level.

Sixth, education level in our sample was higher than the average education level in the Netherlands. It is not clear whether and how this high education level may have influenced the results, but it does mean that we must be careful with generalizing our findings. The same applies to the selection bias, although one might assume that with a smaller selection bias (i.e. higher burnout scores and, thus, a larger sample of people with non-clinical burnout) the effects found would have been larger.

### Suggestions for further research

A primary recommendation for further research is to further investigate the role of working memory in non-clinical burnout, notably the Central Executive component, where the operationalization of the measurement could be further developed. Two issues are of importance here. First, one could add a shifting and inhibition condition to enhance the task complexity (and thus gain more insight in the sensitivity of tests). Second, combining the Letter-Number Sequencing test with the Digit Span Tests will likely give a more precise measurement of verbal working memory.

Researchers following-up on this study, for example, with a larger sample or a longitudinal design, should keep in mind the importance of cognitive functioning in relation to work performance and sleep quality.

In follow-up studies it would be a good addition if sleep is also objectively determined for example by polysomnography or with more versatile and lesser invasive methods, such as wearable devices.

Both depressive and anxiety disorders affect cognition ([82]), work performance [83] and sleep [84] with depressive disorder having a greater effect in all three cases. Given the larger impact of depression and the limited length of our test session, we decided to include just a depression questionnaire. Future studies may wish to expand to include measurements of anxiety disorders as well.

In our study we focused on insomnia, which is just one aspect of sleep quality. In future studies, other dimensions of sleep quality, for instance sleep duration and sleep regularity, are also of importance to investigate.

Finally, a combination of neuropsychological testing with neuroendocrinological measures or brain imaging might yield important insights into non-clinical burnout and cognitive functioning.

## Conclusion

Our results indicate that non-clinical burnout is associated with more sleep problems (but not necessarily insomnia), more depressive complaints (but not necessarily depression), impaired work performance, and with both subjective and objective cognitive impairments. Compared with healthy respondents (*N* = 107), people with non-clinical burnout (*N* = 17) had a significantly poorer performance on the tests of the visuospatial sketchpad (Corsi Block Test) and the Central Executive (with dual task performance) of working memory. Our study suggests that more complex (dual) tests are more sensitive in detecting non-clinical burnout. Dual-task performance was significantly related to work performance. In our sample of people with non-clinical burnout, there were no severe sleep problems. Further analysis of the entire sample, however, revealed that insomnia was significantly related with subjective cognitive functioning, but not with objective cognitive functioning or work performance. Adaptation of the workplace with reduction of task-switching and cognitive training in non-clinical burnout might be viable preventive interventions. Further research with larger samples and a more extensive operationalization of measurement should be carried out to further establish our findings.
